# The *κ*-Deformed Calogero–Leyvraz Lagrangians and Applications to Integrable Dynamical Systems

**DOI:** 10.3390/e24111673

**Published:** 2022-11-17

**Authors:** Partha Guha

**Affiliations:** Department of Mathematics, Khalifa University of Science and Technology, Abu Dhabi P.O. Box 127788, United Arab Emirates; partha.guha@ku.ac.ae

**Keywords:** entropic kinetic energy, Lotka–Volterra, replicator equation, relativistic Toda lattice, Kaniadakis logarithm, Tsallis deformation, *κ*-deformed Lagrangian, 34C14, 34C20

## Abstract

The Calogero–Leyvraz Lagrangian framework, associated with the dynamics of a charged particle moving in a plane under the combined influence of a magnetic field as well as a frictional force, proposed by Calogero and Leyvraz, has some special features. It is endowed with a Shannon “entropic” type kinetic energy term. In this paper, we carry out the constructions of the 2D Lotka–Volterra replicator equations and the N=2 Relativistic Toda lattice systems using this class of Lagrangians. We take advantage of the special structure of the kinetic term and deform the kinetic energy term of the Calogero–Leyvraz Lagrangians using the κ-deformed logarithm as proposed by Kaniadakis and Tsallis. This method yields the new construction of the κ-deformed Lotka–Volterra replicator and relativistic Toda lattice equations.

## 1. Introduction

Recently, Calogero and Leyvraz [1,2] demonstrated a time-independent Hamiltonian description of the motion of a charged particle moving in a two dimensional space under the influence of a magnetic field perpendicular to the plane of motion and a frictional force proportional to the velocity. This motion may be viewed as a dynamics of cyclotron motion with friction; this model arises through the coupling of a particle to a large number of external degrees of freedom. The most interesting feature of this Lagrangian is the kinetic energy term—entropic type kinetic energy. This makes the Lagrangian a nonstandard one. We have explored the applications of this class of Lagrangians in our earlier papers [3,4].

It is worth noting that some physical systems cannot be described by the Boltzmann–Gibbs statistical mechanics, for example, systems such as long-range interactions, long-time memory and multifractal or hierarchical structures are some of them. To overcome at least some of these difficulties, Tsallis [5,6] proposed a generalized entropic form based on a κ-deformed logarithm. Later, an example of self-dual κ-deformed logarithmic functions is found in the work of Kaniadakis [7,8,9,10,11]. Over the last decade or so, scientists have observed that many physical and social phenomena often follow the so-called power law distributions (see for example, [12,13,14,15]). We demonstrated that many (generalized) power law distribution equations can be derived from Calogero–Leyvraz Lagrangian formalism using κ-deformation theory.

These are the two popular ways to deform logarithmic and exponential terms in physics. We propose a new Lagrangian where the logarithm term appearing in Calogero–Leyvraz is replaced by a deformed logarithmic function, and study the dynamics. The algebraic structures arising in this κ-deformed framework have been carefully grafted by Scarfone in [16]; in fact, the concept of generalized algebras has been employed constructively to study entropic forms in [16,17]. It is worth noting that the generalized entropies [18] play an important role in generalized distribution theory in complex systems [19] and they have been studied extensively from the information geometric point of view in [20,21]. The aim of this work is to carry out the formulation of the κ-deformed well-known dynamical systems, namely, the Lotka–Volterra replicator equation and N=2 relativistic Toda lattice system.

Among ecological models, the Lotka–Volterra equation for predator–prey systems [22,23,24] has played a significant role in dynamical systems. It is known that the solutions to this conservative system in phase space are level curves of the energy function. In the mathematical investigations of ecological models, conservative dynamics are often very useful from the (geometrical) mechanics point of view. It is known that the replicator equation in the evolutionary game theory [25] is closely related to the Lotka–Volterra equation. The replicator equation is the first and most important game dynamics studied in connection with evolutionary game theory. It was originally developed for symmetric games with finitely many strategies. Evolutionary game theory [26] studies the behavior of large populations of agents who repeatedly engage in strategic interactions. Note that changes in behavior in these populations are driven either by natural selection via differences in birth and death rates, or by the application of myopic decision rules by individual agents.

The Hamiltonian of the two-dimensional motion of electrons in the presence of the periodic potential and the magnetic field perpendicular to the two-dimensional plane is described by:

(1) $$H_{Hof}=e^{iq}+e^{-iq}+e^{ip}+e^{-ip}\;\left[q,p\right]=i\hbar .$$

The spectrum of this system yields a butterfly like structure, known as Hofstadter’s butterfly [27]. In a completely independent line of research, the string community investigated a system associated to Hamiltonian,

(2) $$H=e^{q}+e^{-q}+e^{p}+e^{-p},$$

when *q* and *p* are restricted to be purely imaginary; this equation reduces to Hofstadter’s Hamiltonian (1). In general, *q* and *p* are complex coordinates, hence the equation determines a real two-dimensional Riemann surface, or equivalently a complex one-dimensional curve, whose shape is parameterized by the value of *H* [28,29]. This appears when mirror symmetry is applied to a non-compact Calabi–Yau geometry known as the local $P_{1}\times P_{1}$ geometry. This curve is connected to the Seiberg–Witten curve, encoding the information on instantons in N=2 supersymmetric pure SU(2) gauge theory [30,31,32].

The system known nowadays as the relativistic Toda lattice (RTL) was invented by S.N.M. Ruijsenaars—the Hamiltonian of the periodic relativistic Toda lattice with just N=2 particle, after removing the center-of-mass mode. We can illustrate this as follows [28,29]. The Ruijsenaars Hamiltonian [33] of N=2 quantum Toda lattice is given by:

(3) $$H_{RT}=e^{Rp_{1}}+e^{Rp_{2}}+R^{2}(e^{q_{1}-q_{2}+Rp_{1}}+e^{q_{2}-q_{1}+Rp_{2}}).$$

Let us consider center of mass frame $p_{1}+p_{2}=0$ and define

$$p:=Rp_{1},\;q:=q_{1}-q_{2}+Rp_{1},$$

which yields an one parameter family of (2),

(4) $$H_{RToda}=e^{p}+e^{-p}+R^{2}\left(e^{q}+e^{-q}\right).$$

**Results of the paper** The Calogero–Leyvraz Lagrangian has some interesting features; it is endowed with the Shannon entropic [34] kinetic energy term. The Legendre transform of this Lagrangian yields a Hamiltonian with the exponential momentum term. We have seen that the Calogero–Leyvraz Lagrangian/Hamiltonian allows us to formulate several features related to deformed dynamical systems, balanced loss–gain systems and generalized rate equations [3,4]. In particular, most of the power law distributions and rate equations can be manufactured from this class of Lagrangian. It has been explored that the Calogero–Leyvraz theory of cyclotron-friction motion is closely related to the “curl force” theory as proposed by Berry and Shukla [35,36], although the latter is a totally position-dependent nonconservative force with a nonvanishing curl, whereas the former is totally velocity dependent.

In this paper, we present a different formulation of the celebrated Lotka–Volterra equation [22,24] using the Calogero–Leyvraz Lagrangian. We also give a new derivation of the replicator equation using the Calogero–Leyvraz type Lagrangian. The final example is related to the N=2 relativistic Toda lattice system. We formulate the latter equation using a different type of entropic Lagrangian; this entropic kinetic energy is described via a cross-entropy term, which yields a new formulation of the N=2 relativistic Toda lattice system.

Since the kinetic energy term of the Calogero–Leyvraz Lagrangian involves a logarithmic term, we deform this logarithmic term using the Kaniadakis method [17] and obtain κ-deformed Lotka–Volterra, replicator and N=2 relativistic Toda Lattice equations. In fact, the entire reason to shift the usual formalism to Calogero–Leyvraz formalism is to formulate κ-deformed integrable models. We also formulate these deformed equations using the Tsallis logarithm.

In the introductory section, we review a κ-deformed Liénard equation which satisfies the Chiellini integrability condition. This condition allows us to integrate the Liénard type equation using the Abel equation of the first kind. In general, the Liénard equation does not give a Lagrangian formulation, but with the imposition of the Chiellini condition, it yields a Lagrangian formulation.

This paper is *organized* as follows. In Section 2, we review the Calogero–Leyvraz Lagrangians and Hamiltonians and their applications. In particular, we also describe the κ-deformed Liénard type equation using Kaniadakis and Tsallis type deformation of the kinetic energy term and demonstrate that this Liénard equation admits the Chiellini integrability condition [37]. This integrability condition plays an important role in the formulation of Lagragian and solutions of the integrable class of the Liénard equation. A nonexhaustive list of applications includes, among others, those in [38,39,40]. After giving a gentle introduction to the Calogero–Leyvraz method, we apply this scheme for the construction of the Lotka–Volterra, replicator and N=2 relativistic Toda lattice systems in Section 3. Our Section 4 is dedicated to the construction of κ-deformed equations. We give a formulation of the deformed Lotka–Volterra, replicator and N=2 relativistic Toda lattice equations using the Kaniadakis and Tsallis methods.

## 2. Review of Calogero–Leyvraz’s Lagrangian and Hamiltonian Formulation of the Dynamics of Cyclotron with Friction System

The Hamiltonian of the free particle moving against friction is given by:

(5) $$H\left(p,z\right)=e^{p}+cz,$$

according to the Newtonian equation of motion $\ddot{z}=-\dot{z}$. The corresponding Lagrangian description of this system is given by:

(6) $$L=\dot{z}\ln\;\dot{z}-cz.$$

A minor modification of the Hamiltonian $H\left(p,z\right)=e^{p}+\frac{\lambda p}{c}+cz$ yields the dynamics of a particle moving against friction in a constant force field λ.

**Legendre transformation of Calogero–Leyvraz Lagrangian:** Let us recall the Calogero–Leyvraz method first; the Lagrangian is given as L=−γq+vlnv, where $v=\dot{q}$. The equation of motion $\ddot{q}+\gamma \dot{q}=0$ yields a constant of motion

(7) $$C=v+\gamma q,\;\;\mathrm{where}\;\;v=\dot{q}.$$

The corresponding momentum

$$p=\frac{\partial L}{\partial v}=\left(\ln v+1\right)\;\;\;\Rightarrow \;\;\;v=e^{p-1}.$$

Substituting this in the Legendre transformation,

(8) FL(L)=vp−L=v(1+lnv)−L=v+γq;

thus we obtain the Calogero–Leyvraz Hamiltonian,

(9) $$H_{CL}=e^{p}+\gamma q,$$

where we have scaled the momentum to ignore the constant term. Hence we establish the connection between the Calogero–Leyvraz Lagrangian and Hamiltonian via Legendre transformation. This construction can be extended to Lagrangian involving a time-dependent coefficient.

### 2.1. Calogero–Leyvraz Hamiltonian and Planar Systems

Calogero and Leyvraz straight-forwardly generalized this motion by complexification to describe motions taking place in a plane. Physically, this is connected to the motion against friction of a charged particle in the presence of a perpendicular constant magnetic field, or a constant electric field lying in that plane, or both these forces. If we set c=γ+iω and go to the complex plane, the following pair of Poisson commuting Hamiltonians are obtained:

$$H_{R}=e^{p_{x}}\cos p_{y}+\gamma x-\omega y,\;H_{I}=-e^{p_{x}}\sin p_{y}+\omega x+\gamma y.$$

At first we consider a minor change; coefficients are considered to be time dependent. The two-dimensional Calogero–Leyvraz model is given by the following Hamiltonian:

(10) $$H=e^{p_{x}}\cos p_{y}+\gamma \left(t\right)x-\omega \left(t\right)y,$$

where γ(t),ω(t) are parameters, (x,y) are coordinates and $\left(p_{x},p_{y}\right)$ are corresponding momenta. Here we note that the potential energy is a linear function of coordinates while the kinetic energy $\Psi =e^{p_{x}}\cos p_{y}$. The Hamiltonian (10) yields the following equations of motion:

(11) $$\ddot{x}=-\gamma \left(t\right)\dot{x}+\omega \left(t\right)\dot{y},\;\ddot{y}=-\gamma \left(t\right)\dot{y}-\omega \left(t\right)\dot{x}.$$

Calogero and Leyvraz reformulated (11) in a 3-dimensional context by introducing the 3-vector r=(x,y,0) in the xy-Cartesian plane and the unit vector $\hat{z}=\left(0,0,1\right)$ orthogonal to that plane; this yields:

(12) $$\ddot{r}=-\gamma \left(t\right)\dot{r}+\omega \left(t\right)\dot{r}\times \hat{z}.$$

We obtain the sister (or mirror) equations of (11) if we consider a different K.E., viz., $\Phi \left(p_{x},p_{y}\right)=e^{p_{x}}\sin p_{y}$, with the same potential energy γx−ωy, this is given by:

(13) $$\ddot{x}=-\gamma \left(t\right)\dot{x}+\omega \left(t\right)\dot{y},\;\ddot{y}=\gamma \left(t\right)\dot{y}-\omega \left(t\right)\dot{x}.$$

It is easy to check both (11) and (13).

The linear equation can be generalized to a nonlinear equation from the Calogero–Leyvraz Hamiltonian using generalized potential energy. Suppose we consider

(14) $$H=e^{p_{x}}\cos p_{y}+\gamma \left(t\right)\phi \left(x,y\right)-\omega \left(t\right)\psi \left(x,y\right),$$

where ϕ and ψ are some functions of *x* and *y*. This yields

(15) $$\ddot{x}=-\gamma \left(t\right)\phi_{x}\left(x,y\right)\dot{x}+\omega \left(t\right)\psi_{y}\left(x,y\right)\dot{y},\;\ddot{y}=-\gamma \left(t\right)\phi_{x}\left(x,y\right)\dot{y}-\omega \left(t\right)\psi_{y}\left(x,y\right)\dot{x}.$$

The complex kinetic energy is given by:

(16) $$K_{c}=\Psi +i\Phi =e^{p_{x}}\cos p_{y}+ie^{p_{x}}\sin p_{y}=e^{p_{x}+ip_{y}}=e^{P},\;P=p_{x}+ip_{y}.$$

If we consider the (complex) potential energy $U_{c}=\Gamma \left(t\right)z$, then the equation of motion resulting from the Hamiltonian $H=e^{P}+\Gamma z$ is

(17) $$\ddot{z}=\Gamma \left(t\right)\dot{z}.$$

Suppose Re(P)=p and Re(z)=a(t)x, then the Hamiltonian becomes $H_{R}=e^{p}+q$ and this yields $\ddot{x}=-a\left(t\right)\dot{x}$. Note that a nonlinear potential energy with the same K.E. yields nonlinear equations, for example, $H_{1}=e^{p}+a\left(t\right)\ln\;x$ and $H_{2}=e^{p}+a\left(t\right)x^{n}$ yield the following equations of motion: *viz*

(18) $$x\ddot{x}+a\left(t\right)\dot{x}=0,\;\ddot{x}+na\left(t\right)x^{n-1}\dot{x}=0.$$

respectively.

Equation (17) admits, for constant Γ, a Lagrangian:

(19) $$L\left(z,\dot{z}\right)=-z+\Gamma^{-1}\dot{z}\log\dot{z},$$

with (17) following from the associated Euler–Lagrange equation.

### 2.2. Illustration: Generalized Liénard Equation and the Calogero–Leyvraz Lagrangian

We have seen that the Calogero and Leyvraz construction yields interesting sets of dynamical equations. In this section, we formulate a nonlinear ODE belonging to the Liénard class of equations.

The Liénard type ordinary second order nonlinear differential equation is given by:

(20) $$\ddot{q}+f\left(q\right)\dot{q}+g\left(q\right)=0.$$

*f* and *g* are two continuously differentiable functions on R. Since the Liénard equation itself is also an autonomous differential equation, the substitution, $y=\frac{dq}{dt}$ or q=∫y(t)dt, leads the Liénard equation to become a first order differential,

(21) $$y\frac{dy}{dq}+f\left(q\right)y+g\left(q\right)=0,$$

which belongs to the Abel equation of the second kind. This can also be expressed in terms of the Abel equation of the first kind, which we use later.

Let us define the following generalization of the Calogero–Leyvraz Lagrangian:

(22) $$L=\left(\dot{q}+f\left(q\right)\right)\ln\left(\dot{q}+f\left(q\right)\right)-f\left(q\right).$$

The Euler–Lagrange equation yields:

(23) $$\ddot{q}+f^{\prime }\dot{q}+f^{\prime }\ln\left(\dot{q}+f\left(q\right)\right)=0.$$

For small values of $\left(\dot{q}+f\left(q\right)\right)$, the above equation reduces to the well-known nonstandard Lagrangian,

(24) $$\ddot{q}+2f^{\prime }\dot{q}+ff^{\prime }\left(q\right)=0.$$

For different choices of *f* we get different types of equations. Let $f\left(q\right)=\lambda q^{n}$, then n=1; this becomes a damped oscillator equation, n=2; this maps to a second Riccati or modified Emden–Fowler equation. The corresponding Lagrangian is given by:

(25) $$L_{R}=\ln\left(\dot{q}+f\left(q\right)\right).$$

### 2.3. Deformations of Calogero–Leyvraz Lagrangians and κ-Deformed Oscillator Equations

The most attractive feature of the Calogero–Leyvraz Lagrangian is the involvement of a logarithmic term. We grab this opportunity and deform the (entropic) kinetic energy term. We use primarily Kaniadakis and Tsallis logarithms. At first we deform (22) using the Tsallis logarithm.

Let us introduce the Tsallis logarithm. We assume q>0 for practical purposes. The Tsallis *q*-logarithm and *q*-exponential functions are defined by:

(26) $$\ln_{q}\left(x\right)=\frac{x^{1-q}-1}{1-q},\;\exp_{q}\left(x\right)={\left(1+(1-q)x\right)}^{\frac{1}{1-q}},$$

where q≠1 and 1+(1−q)x≠0. For q→1

$$(1+\frac{x}{N}{)}^{N}\approx e^{x},\;N\left(x^{\frac{1}{N}}-1\right)\approx \ln\;x.$$

**Proposition** **1.** *Let $\ln_{\kappa }\left(\dot{q}+f\left(q\right)\right)$ be the Tsallis κ-deformed logarithm. The Euler–Lagrange equation of the Lagrangian*

(27) $$L=\left(\dot{q}+f\left(q\right)\right)\ln_{\kappa }\left(\dot{q}+f\left(q\right)\right)-\mu f\left(q\right),\;-1<\kappa <1,$$

*yields*

(28) $$\left(\kappa +1\right)\left(\left(\ddot{q}+f^{\prime }\dot{q}\right)-\frac{1}{\kappa }f^{\prime }\left(q\right)\left(\dot{q}+f\left(q\right)\right)\right)-\frac{1}{\kappa }\Lambda f^{\prime }\left(q\right){\left(\dot{q}+f\left(q\right)\right)}^{1-\kappa }=0,$$

*where $\Lambda =\mu \frac{\kappa }{\kappa +1}-1$.*

**Proof.**  By direct computation we obtain:

$$\frac{d}{dt}\left(\frac{\partial L}{\partial \dot{q}}\right)={\left(\kappa +1\right)}^{2}\left(\ddot{q}+f^{\prime }\dot{q}\right)(\dot{q}+f\left(q\right){)}^{\kappa },$$

$$\frac{\partial L}{\partial q}=f^{\prime }\left(q\right)\left(\left(1+\kappa \right){\left(\dot{q}+f\left(q\right)\right)}^{\kappa 1}+\ln_{\kappa }(\dot{q}+f\left(q\right)\right)+\mu f^{\prime }\left(q\right)$$

$$=\frac{\kappa +1}{\kappa }f^{\prime }\left(q\right)\left(\left(1+\kappa \right){\left(\dot{q}+f\left(q\right)\right)}^{\kappa }-1+\mu \frac{\kappa }{\kappa +1}\right).$$

Here, we have tacitly used the formula of $\ln_{\kappa }x$. □

**Corollary** **1.** *Suppose $\mu =\frac{\kappa +1}{\kappa }$. The Euler–Lagrange equation of the Lagrangian*

(29) $$L=\left(\dot{q}+f\left(q\right)\right)\ln_{\kappa }\left(\dot{q}+f\left(q\right)\right)-\frac{\kappa +1}{\kappa }f\left(q\right),\;-1<\kappa <1,$$

*yields the*

(30) $$\ddot{q}+\frac{\kappa -1}{\kappa }f^{\prime }\left(q\right)\dot{q}-\frac{1}{\kappa }f^{\prime }f=0.$$

Let us demonstrate this with a couple of examples. Suppose we take $\kappa =\frac{1}{2}$, then (28) reduces to

(31) $$\frac{3}{2}(\ddot{q}-f^{\prime }\left(q\right)\dot{q}-2f^{\prime }f\left(q\right))-2\Lambda f^{\prime }\left(q\right)\sqrt{\left(\dot{q}+f\left(q\right)\right)}=0.$$

If we take $f\left(q\right)={\frac{a}{q}}^{2}$, then Equation (30) yields the second-order Riccati ( also known as the modified Emden ) equation,

(32) $$\ddot{q}+a\frac{\kappa -1}{\kappa }q\dot{q}-\frac{1}{\kappa }a^{2}q^{3}=0.$$

### 2.4. Kaniadakis κ-Deformed Lagrangian, Liénard Equation and Chiellini Integrability Condition

We wish to repeat this calculation using the Kaniadikis κ-deformed logarithm. We obtain the following result. Let $\ln_{\kappa }q=\frac{1}{2\kappa }\left(q^{\kappa }-q^{-\kappa }\right)$ be the Kaniadikis logarithm.

**Proposition** **2.** *The Euler–Lagrange equation for the Lagrangian $L=\left(\dot{q}+f\left(q\right)\right)\ln_{\kappa }\left(\dot{q}+f\left(q\right)\right)-\mu f\left(q\right)$, yields*

(33) $$\begin{array}{cc} \left(\ddot{q}+f^{\prime }\dot{q}-\frac{\kappa }{\kappa +1}f^{\prime }\left(q\right)\right)-\frac{\kappa -1}{\kappa +1}(\ddot{q}+f^{\prime }\dot{q}\\ -\frac{\kappa }{\kappa -1}f^{\prime }\left(q\right)){\left(\dot{q}+f\left(q\right)\right)}^{-2\kappa }+\frac{2\kappa \mu }{\kappa +1}f^{\prime }\left(q\right){\left(\dot{q}+f\left(q\right)\right)}^{-\kappa +1} & =0. \end{array}$$

Suppose we take $\kappa =\frac{1}{2}$ and $f\left(q\right)=\frac{3}{2}q^{2}$, then (33) reduces to

(34) $$\ddot{q}+3q\dot{q}-q+(\ddot{q}+3q\dot{q}+3q){\left(\dot{q}+\frac{3}{2}q^{2}\right)}^{-1}+2q\sqrt{\dot{q}+\frac{3}{2}q^{2}}=0.$$

Consider the κ-deformed Lagrangian without the potential μ term.

**Corollary** **2.** *The Euler–Lagrange equation corresponding to the Lagrangian $L_{\kappa }$ yields a one parameter family of second-order equations,*

(35) $$\begin{array}{cc} \left(1+\kappa \right)\left(\ddot{q}+f^{\prime }\left(q\right)\dot{q}\right)+\left(1-\kappa \right)\left(\ddot{q}+f^{\prime }\left(q\right)\dot{q}\right){\left(\dot{q}+f\left(q\right)\right)}^{-2\kappa }\\ -\frac{f^{\prime }\left(q\right)}{\kappa }\left(\dot{q}+f\left(q\right)\right)\left(\left(1+\kappa \right)-\left(1-\kappa \right){\left(\dot{q}+f\left(q\right)\right)}^{-2\kappa }\right) & =0. \end{array}$$

*This equation is a fractional damped system except for $\kappa =\pm \frac{n}{2}$, where n∈Z.*

The Liénard type ordinary second order nonlinear differential equation can be mapped to the first kind first order Abel differential equation,

(36) $$\frac{dy}{dq}=f\left(q\right)y^{3}+g\left(q\right)y^{2}.$$

This Abel equation allows us to find some exact general solutions of the Liénard type equations by using the integrability conditions of the Abel equation.

**Lemma** **1.** *A first kind Abel type differential equation of the form (36) can be exactly integrated if the functions q(x) and p(x) satisfy the condition:*

(37) $$\frac{d}{dq}\left(\frac{g(q)}{f(q)}\right)=\mu f\left(q\right),\;\mu =\;\mathrm{constant},\;\mu \neq 0.$$

**Claim** **1.** *Equation (35) reduces to the Liénard equation for κ=−1,*

(38) $$\ddot{q}+\frac{1}{2}f^{\prime }\left(q\right)\dot{q}-\frac{1}{2}f^{\prime }\left(q\right)f\left(q\right)=0,$$

*which satisfies the Chiellini condition.*

One can also readily verify that for κ=−1/2, Equation (35) satisfies the generalized Liénard equation,

(39) $$F\left(q,\dot{q}\right)\ddot{q}+2f^{\prime }\left(q\right)\dot{q}-f^{\prime }f\left(3f+2\right)=0,$$

where $F\left(q,\dot{q}\right)=\left(1+\dot{q}+3f\left(q\right)\right)$.

## 3. Entropic Lagrangian and Integrable Class of Systems

In this section, at first we give a new derivation of Lotka–Volterra and replicator equations using Calogero–Leyvraz Lagrangians endowed with the “Shannon entropic” [34] type kinetic energy terms. Then, using the cross entropy type kinetic energy term, we derive the N=2 relativistic Toda lattice equation.

### 3.1. Calogero–Leyvraz Lagrangian and Lotka–Volterra Equation

We start with the derivation of the Lotka–Volterra equation. Consider the following logarithmic Lagrangian endowed with an entropic kinetic term:

(40) $$L=\left(1-\dot{q}\right)\ln\;\left(1-\dot{q}\right)-aq-ae^{-q}.$$

The Euler–Lagrange equation yields:

(41) $$\frac{\ddot{q}}{1-\dot{q}}+1-e^{-q}=0.$$

Let us write this equation as a system of first-order equations. Define:

(42) $$1-\dot{q}=z,\;e^{-q}=y.$$

Then (41) equation can recasted as:

(43) $$\dot{y}=\left(zy-y\right),\;\dot{z}=a\left(z-zy\right).$$

This is a standard form of the celebrated 2D Lotka–Volterra equation in non-dimensionalized form [23].

In the standard formalism of the Lotka–Volterra equation (43), the Hamiltonian is given by:

(44) H=z−lnz+ay−alny.

The nonstandard Hamiltonian form,

$$\left(\begin{array}{c} \dot{y}\\ \dot{z} \end{array}\right)=\left(\begin{array}{cc} 0 & yz\\ -yz & 0 \end{array}\right)\left(\begin{array}{c} \frac{\partial H}{\partial y}\\ \frac{\partial H}{\partial z}, \end{array}\right)$$

yields Equation (43). The two Hamiltonians can be connected easily through exponential mapping.

### 3.2. Replicator Equation

In 1978, Taylor and Jonker [41] introduced a system of differential equations which were designated later on as the replicator equation. This equation plays an important role in evolutionary game theory. The replicator equation models the frequency evolution of certain strategic behaviors within a biological population. Hofbauer [25] unveiled an equivalence relation between the Lotka–Volterra equation and the replicator equation.

Consider the first population where individuals interact with each other according to a set of n+1 pure strategies $E_{0},\cdots ,E_{n}$ with relative frequencies $x_{0},\cdots ,x_{n}$, and the second population plays different m+1 pure strategies $F_{0},\cdots ,F_{m}$ with frequencies $y_{0},\cdots ,y_{m}$. After a contest $E_{i}$ versus $F_{j}$, the payoff for the first player is $a_{ij}$ whereas for the second player it is $b_{ji}$. Let $A=(a_{ij})$ be the matrix consisting of these $a_{ij}$ and so also *B*, then for such games the evolutionary dynamics is given by:

(45) $$\dot{x_{i}}=x_{i}\left({\left(Ay\right)}_{i}-x\dot{A}y\right),\;i=0,\cdots ,n$$

(46) $$\dot{y_{j}}=y_{j}\left({\left(Bx\right)}_{j}-y\dot{B}x\right),\;j=0,\cdots ,m.$$

Adding or multiplying a (positive) constant to each column of *A* or *B* does not alter the dynamics.

In the case of n=m=1, the above equations simplify to:

(47) $$\dot{x}=x\left(1-x\right)\left(a-(a+b)y\right),\;\dot{y}=y\left(1-y\right)\left(-c+(c+d)x\right).$$

We will use the Calogero–Leyvraz type Lagrangian to derive the planar replicator type equation.

#### Calogero–Leyvraz Lagrangian and Replicator Equation

We define the Lagrangian of the coupled system as:

(48) $$L=\dot{q_{i}}\ln\;\dot{q_{i}}+\left(1-{\dot{q}}_{i}\right)\ln\;\left(1-{\dot{q}}_{i}\right)+\lambda {\dot{q}}_{1}q_{2}-\mu {\dot{q}}_{2}q_{1}+aq_{1}-cq_{2},\;i=1,2.$$

It is straight forward to see that the Lagrangian (48) yields:

(49) $${\ddot{q}}_{1}={\dot{q}}_{1}\left(1-{\dot{q}}_{1}\right)\left(a-\left(\lambda +\mu \right){\dot{q}}_{2}\right),\;{\ddot{q}}_{2}={\dot{q}}_{2}\left(1-{\dot{q}}_{2}\right)\left(-c+\left(\lambda +\mu \right){\dot{q}}_{1}\right).$$

Let us define:

(50) $${\dot{q}}_{1}=x,\;{\dot{q}}_{2}=y.$$

We obtain the replicator equation from (49):

(51) $$\dot{x}=x\left(1-x\right)\left(a-(\lambda +\mu )y\right),\;\dot{y}=y\left(1-y\right)\left(-c+(\lambda +\mu )x\right).$$

### 3.3. Logarithmic Lagrangian Formulation of N=2 Relativistic Toda Lattice Equation

In this example, we consider a Lagrangian with the *cross entropic* type kinetic energy term,

(52) $$L=\dot{q}\ln\;\left(\dot{q}+\sqrt{{\dot{q}}^{2}+1}\right)-(\left(\dot{q}+\sqrt{{\dot{q}}^{2}+1}\right)-\left(\dot{q}+\sqrt{{\dot{q}}^{2}+1}{)}^{-1}\right)-\cosh q.$$

**Proposition** **3.** *The Euler–Lagrange equation, the Lagrangian (52), yields:*

(53) $$\frac{\ddot{q}}{\sqrt{{\dot{q}}^{2}+1}}=-\sinh\;q.$$

**Proof.**  After an elaborate calculation from the entropic K. E. term ($L_{1}$), it yields:

$$\frac{\partial L_{1}}{\partial \dot{q}}=\ln\;\left(\dot{q}+\sqrt{{\dot{q}}^{2}+1}\right)+\frac{\dot{q}}{\sqrt{{\dot{q}}^{2}+1}},$$

and the second K.E. term $(\left(\dot{q}+\sqrt{{\dot{q}}^{2}+1}\right)-\left(\dot{q}+\sqrt{{\dot{q}}^{2}+1}{)}^{-1}\right)=L_{2}$ yields:

$$\frac{\partial L_{2}}{\partial \dot{q}}=\frac{\dot{q}}{\sqrt{{\dot{q}}^{2}+1}}.$$

These two expressions lead to a magical cancellation of the term $\frac{\dot{q}}{\sqrt{{\dot{q}}^{2}+1}}$. Using the Euler–Lagrange equation we obtain the equation. □

The Ruijsenaars Hamiltonian is given by:

(54) $$H=\sum_{n=1}^{N}\left(1+q^{-1/2}R^{2}e^{q_{n}-q_{n+1}}\right)e^{Rp_{n}},$$

where $q=e^{iR\hbar }$.

For N=2 case this equation reduces to:

(55) $$H_{2}=e^{Rp_{1}}+e^{Rp_{2}}+R^{2}\left(e^{q_{1}-q_{2}+Rp_{1}}+e^{q_{2}-q_{1}+Rp_{2}}\right).$$

Consider the centre of mass frame $p_{1}+p_{2}=0$. Let us define:

(56) $$p:=Rp_{1},\;q:=q_{1}-q_{2}+Rp_{1}.$$

We express $H_{2}$ as:

(57) $$\hat{H_{2}}=e^{p}+e^{-p}+R^{2}\left(e^{q}+e^{-q}\right);$$

for practical purposes we scaled R=1.

Let us express Hamiltonian (57) in terms of cosine hyperbolic function

(58) H=coshp+coshq,

where we drop the factor 2. The Hamiltonian equation yields:

(59) $$\dot{q}=\frac{\partial H}{\partial p}=\sinh\;p,\;\dot{p}=-\frac{\partial H}{\partial q}=-\sinh\;p,$$

which reduces to Equation (53).

#### 3.3.1. Connection to Calabi–Yau Manifold

We must note that the energy function $E=e^{p}+e^{-p}+e^{q}+e^{-q}$ can be expressed as:

(60) $$X+X^{-1}+Y+Y^{-1}=E.$$

This defines a genus one Riemann surface. The complex 3D space $V=X+X^{-1}+Y+Y^{-1}-E$ describes a Calabi–Yau manifold. This sets up a connection with the Calabi–Yau manifold. The Riemann surface has enough information to describe this Calabi–Yau manifold. The energy function E(p,q) considered to be Hamiltonian appears in the string theory.

Mirror symmetry states that a CY manifold has its mirror dual. The Kähler structure of the original CY is mapped to the complex structure of the mirror CY, and vice versa. In our case, the mirror curve is given by:

(61) $$e^{p}+\mu_{1}e^{-p}+e^{q}+\mu_{2}e^{-q}=1,$$

where $\mu_{1}$ and $\mu_{2}$ are the complex moduli of the mirror CY.

The new equation is in the same form as the Lagrangian of the N=2 relativistic Toda lattice equation. Our case is similar to the case of the quantized mirror curve for the local $P_{1}\times P_{1}$, which is related to the quantum eigenvalue problem of the relativistic Toda lattice with just two particles.

#### 3.3.2. Calogero–Leyvraz Type Lagrangian with Coupling Constant and Mirror Map

Consider the following map:

(62) $$L=\dot{q}\ln\;\left(\dot{q}+\sqrt{{\dot{q}}^{2}+1}\right)-(\left(\dot{q}+\sqrt{{\dot{q}}^{2}+1}\right)-\left(\dot{q}+\sqrt{{\dot{q}}^{2}+1}{)}^{-1}\right)-\gamma^{-1}\cosh q,$$

where $\gamma^{-1}$ is a coefficient ( or coupling ) parameter. The corresponding Hamiltonian or energy function is given as:

(63) $$E=\left(e^{p}+e^{-p}\right)+\gamma^{-1}\left(e^{q}+e^{-q}\right).$$

This yields:

$$\frac{1}{E}=\frac{1}{E^{2}}\left(e^{p}+e^{-p}\right)+\frac{1}{\gamma E}\left(e^{q}+e^{-q}\right).$$

Let us change the variable,

(64) $$e^{p}\mapsto \frac{e^{p}}{E},\;e^{q}\mapsto \frac{\gamma^{-1}}{E}e^{q}.$$

## 4. The κ-Deformed 2D Lotka–Volterra, Replicator and Relativistic Toda Lattice Equations

By deforming the natural logarithm and exponential functions we present κ-deformed 2D Lotka–Volterra and relativistic Toda lattice equations in this section. We will use both Kaniadakis and Tsallis deformations to derive new sets of κ-deformed systems.

### 4.1. The κ Deformation of 2D Lotka–Volterra Equation

We describe two types of κ-deformed systems, semi-deformation and full deformation. In the first case we only deformed the kinetic part, whereas in the second case we consider both the kinetic energy (K.E.) and potential energy (P.E.) parts of the deformations.

**Case 1:** (**deforming only K.E**) Consider the following κ-deformed Lagrangian:

(65) $$L_{\kappa }=\left(1-\dot{q}\right)\ln_{\kappa }\;\left(1-\dot{q}\right)-q-e^{-q}.$$

**Proposition** **4.** *The Euler–Lagrange equation of the deformed Lagrangian (65) reads:*

(66) $$\frac{\ddot{q}}{1-\dot{q}}Exp_{\kappa }\ln\left(1-\dot{q}\right)+1-e^{-q}=0.$$

**Proof.**  It is easy to see that:

$$\frac{\partial L_{\kappa }}{\partial \dot{q}}=-\ln_{\kappa }\;\left(1-\dot{q}\right)-\frac{1}{2}\left({\left(1-\dot{q}\right)}^{\kappa }+{\left(1-\dot{q}\right)}^{-\kappa }\right),$$

$$\;=-\frac{1}{\kappa }\sinh\;\kappa \ln\left(1-\dot{q}\right)-\cosh\kappa \ln\left(1-\dot{q}\right).$$

Thus we obtain:

$$\frac{d}{dt}\left(\frac{\partial L_{\kappa }}{\partial \dot{q}}\right)=\frac{\ddot{q}}{1-\dot{q}}\left(\cosh\kappa \ln\left(1-\dot{q}\right)+\kappa \sinh\;\kappa \ln\left(1-\dot{q}\right)\right)=\frac{\ddot{q}}{1-\dot{q}}Exp_{\kappa }\ln\left(1-\dot{q}\right),$$

where the generalized κ deformed exponential is given by

(67) $$Exp_{\kappa }x=\cosh\kappa x+\kappa \sinh\kappa x.$$

The final result follows from the remaining part of the calculation. □

It is clear that when κ→0 we recover the ordinary Lotka–Volterra equation.

**Case 2:** (**deforming both K.E. and P.E.**) In this case we also deformed the exponential term $e^{-q}$ in the potential. We take the following Lagrangian:

(68) $$L_{\kappa }^{d}=\left(1-\dot{q}\right)\ln_{\kappa }\;\left(1-\dot{q}\right)-q-exp_{\kappa }\left(-q\right).$$

Thus we obtain the following result from the straightforward computation.

**Proposition** **5.** *The Euler–Lagrange equation of the deformed Lagrangian $L_{\kappa }^{d}$ yields:*

(69) $$\frac{\ddot{q}}{1-\dot{q}}Exp_{\kappa }ln\;\left(1-\dot{q}\right)+1-\frac{1}{\sqrt{1+\kappa^{2}q^{2}}}exp_{\kappa }\left(-q\right)=0.$$

#### 4.1.1. Expressing κ-Deformed Equation

The inverse of the generalized κ deformed exponential $Exp_{\kappa }\left(x\right)$ is given by:

(70) $$Exp_{\kappa }{\left(x\right)}^{-1}=Exp_{-\frac{1}{\kappa }}\left(x\right)=\cosh\kappa x-\frac{1}{\kappa }\sinh\kappa x.$$

We now express Equation (66) in a standard form. Let us define:

(71) $$1-\dot{q}=z,\;y=e^{-q}.$$

Thus we obtain:

$$\dot{y}=-e^{-q}\dot{q}=-y\left(z-1\right),\;\frac{-\dot{z}}{z}Exp_{\kappa }\ln\;z+1-y=0.$$

This can be expressed as:

(72) $$\dot{y}=y-zy,\;\dot{z}Exp_{\kappa }\ln\;z=z-yz.$$

We recover the original equation when κ→0.

Let w=lnz or $z=e^{w}$. Then the second equation becomes

(73) $$\dot{w}w=Exp_{-\frac{1}{\kappa }}\left(w\right)\left(1-y\right).$$

A further change of variable $p=\frac{1}{2}w^{2}$ yields a modified set of deformed Lotka–Volterra equations:

(74) $$\dot{q}=\left(e^{\sqrt{2p}}-1\right),\;\dot{p}=d\left(1-e^{-q}\right),\;\;\mathrm{where}\;\;\;\;\;d=Exp_{-\frac{1}{\kappa }}\left(\sqrt{2p}\right).$$

Hence we express the deformed Lotka–Volterra equation in a standard form using the generalized κ deformed exponential function.

#### 4.1.2. Tsallis Logarithm and Deformed Lotka–Volterra System

In section we express the Tsallis logarithm and exponential in terms of κ, which are given as:

(75) $$\ln_{\kappa }\left(q\right)=\frac{q^{\kappa }-1}{\kappa },\;\exp_{\kappa }{\left(q\right)=(1+\kappa q)}^{\frac{1}{\kappa }}.$$

We now deform the Lotka–Volterra Lagrangian using the κ-deformed Tsallis logarithm and exponential. It is defined as:

(76) $$L_{\kappa }^{T}=\left(1-\dot{q}\right)\ln_{\kappa }\;\left(1-\dot{q}\right)-q-\exp_{\kappa }\left(-q\right),$$

with

(77) $$\ln_{\kappa }\;\left(1-\dot{q}\right)=\frac{{\left(1-\dot{q}\right)}^{\kappa }-1}{\kappa },\;\exp_{\kappa }\left(-q\right){=(1-\kappa q)}^{\frac{1}{\kappa }},$$

where 1−κq>0.

**Proposition** **6.** *The Euler–Lagrange equation associated with the Tsallis deformed Lagrangian $L_{\kappa }^{T}$ yields:*

(78) $$\ddot{q}\left(1+\kappa \right){\left(1-\dot{q}\right)}^{\kappa -1}+1-\frac{\exp_{\kappa }\left(-q\right)}{\left(1-\kappa q\right)}=0.$$

**Proof.**  Using the properties of the Tsallis logarithm and exponential functions we arrive at our desired result. □

One can readily check that, when κ→0, Equation (79) reduces to the usual Lotka–Volterra equation. If we assume only the deformation of the kinetic term using the Tsallis logarithm, then Equation (79) reduces to:

(79) $$\ddot{q}{\left(1+\kappa \right)}^{2}{\left(1-\dot{q}\right)}^{\kappa -1}+1-\exp\left(-q\right)=0.$$

### 4.2. The κ-Deformed Replicator Equation

We can deform the Lagrangian of the replicator Equation (48) using the Kaniadakis or Tsallis deformation of logarithm term. Using Kaniadakis deformation we obtain:

(80) $$L=\dot{q_{i}}\ln_{\kappa }\;\dot{q_{i}}+\left(1-{\dot{q}}_{i}\right)\ln\;\left(1-{\dot{q}}_{i}\right)+\lambda {\dot{q}}_{1}q_{2}-\mu {\dot{q}}_{2}q_{1}+aq_{1}-cq_{2},\;i=1,2.$$

**Proposition** **7.** *With the Euler–Lagrange equation related to the deformed Lagrangian (80), we obtain the Kaniadakis deformed coupled equation:*

(81) $$\ddot{q_{1}}\left(\left(1-\dot{q_{1}}\right)\cosh\;\kappa \ln\;\dot{q_{1}}+\dot{q_{1}}\cosh\;\kappa \ln\;\left(1-{\dot{q}}_{1}\right)\right)=\dot{q_{1}}\left(1-\dot{q_{1}}\right)\left(a-\left(\lambda +\mu \right){\dot{q}}_{2}\right),$$

(82) $$\ddot{q_{2}}\left(\left(1-\dot{q_{2}}\right)\cosh\;\kappa \ln\;{\dot{q}}_{2}+{\dot{q}}_{2}\cosh\;\kappa \ln\;\left(1-\dot{q_{2}}\right)\right)\left(-c+\left(\lambda +\mu \right){\dot{q}}_{1}\right).$$

**Corollary** **3.** *The Kaniadakis κ deformed replicator equation is given by:*

(83) $$\dot{x}\left((1-x)\cosh\;\kappa \ln\;x+x\cosh\;\kappa \ln\;(1-x)\right)=x\left(1-x\right)\left(a-(\lambda +\mu )y\right),$$

(84) $$\dot{y}\left((1-y)\cosh\;\kappa \ln\;y+y\cosh\;\kappa \ln\;(1-y)\right)=y\left(1-y\right)\left(-c+(\lambda +\mu )x\right),$$

*where $x={\dot{q}}_{1}$ and $y={\dot{q}}_{2}$.*

One can readily see when κ→0 the deformed replicator Equations (83) and (84) reduces to the original replicator equation.

We can repeat the same procedure using Tsallis deformation of the logarithm. The coupled equations are given by:

(85) $$\left(1+\kappa \right){\ddot{q}}_{1}\left({\dot{q}}_{1}^{\kappa -1}+{\left(1-{\dot{q}}_{1}\right)}^{\kappa -1}\right)=a-\left(\lambda +\mu \right){\dot{q}}_{2},\;\left(1+\kappa \right){\ddot{q}}_{2}\left({\dot{q}}_{2}^{\kappa -1}+{\left(1-{\dot{q}}_{2}\right)}^{\kappa -1}\right)=-c+\left(\lambda +\mu \right){\dot{q}}_{1},$$

which leads to the Tsallis deformed replicator equation,

(86) $$\left(1+\kappa \right)\dot{x}\left(x^{\kappa -1}+{\left(1-x\right)}^{\kappa -1}\right)=a-\left(\lambda +\mu \right)y,\;\left(1+\kappa \right)\dot{y}\left(y^{\kappa -1}+{\left(1-y\right)}^{\kappa -1}\right)=-c+\left(\lambda +\mu \right)x.$$

This again reduces to the original replicator equation for κ→0.

### 4.3. The κ-Deformed N=2 Relativistic Toda Lattice System

In this section, at first we also modify the entropic kinetic energy term, keeping all other terms unchanged. The Kaniadakis κ-deformed Lagrangian for the N=2 relativistic Toda lattice system is defined as:

(87) $$L_{1\kappa }=\dot{q}\ln_{\kappa }\left(\dot{q}+\sqrt{{\dot{q}}^{2}+1}\right)-\frac{1}{2}\left(\dot{q}+\sqrt{{\dot{q}}^{2}+1})+\frac{1}{\dot{q}+\sqrt{{\dot{q}}^{2}+1}}\right)-\gamma^{-1}\cosh\;q.$$

**Proposition** **8.** *The Euler–Lagrange equation corresponding to the κ-deformed Lagrangian $L_{1\kappa }$ yields:*

(88) $$\frac{\ddot{q}}{\sqrt{1+{\dot{q}}^{2}}}\cosh\;\kappa \ln\;\left(\dot{q}+\sqrt{1+{\dot{q}}^{2}}\right)+\frac{d}{dt}\left(\frac{\dot{q}}{\sqrt{1+{\dot{q}}^{2}}}\left(\cosh\;\kappa \ln(\;\dot{q}+\sqrt{1+{\dot{q}}^{2}})-1\right)\right)+\gamma^{-1}\sinh\;q=0.$$

**Proof.**  This proof follows from the direct computation. □

One can readily check that for κ→0 (89) reduces to the ordinary N=2 relativistic Toda lattice equation.

For the most general case, we deform the potential term coshq too, which yields the following Lagrangian:

$$L{=}_{1\kappa }=\dot{q}\ln_{\kappa }\left(\dot{q}+\sqrt{{\dot{q}}^{2}+1}\right)-\frac{1}{2}\left(\dot{q}+\sqrt{{\dot{q}}^{2}+1})+\frac{1}{\dot{q}+\sqrt{{\dot{q}}^{2}+1}}\right)-\gamma^{-1}\cosh_{\kappa }q.$$

We obtain κ-deformed N=2 relativistic Toda lattice equation,

(89) $$\frac{\ddot{q}}{\sqrt{1+{\dot{q}}^{2}}}\cosh\;\kappa \ln\;\left(\dot{q}+\sqrt{1+{\dot{q}}^{2}}\right)+\frac{d}{dt}\left(\frac{\dot{q}}{\sqrt{1+{\dot{q}}^{2}}}\left(\cosh\;\kappa \ln\;(\dot{q}+\sqrt{1+{\dot{q}}^{2}})-1\right)\right)+\frac{1}{\sqrt{1+{\dot{q}}^{2}}}\gamma^{-1}\sinh_{\kappa }\;q=0.$$

This yields the most general Kaniadakis κ-deformation of the N=2 relativistic Toda lattice equation which reduces to the original one when κ goes to zero.

#### Tsallis Deformed N=2 Relativistic Toda Lattice Equation

In this section we present the deformation of the the N=2 relativistic Toda lattice equation using Tsallis deformation. Let the entropic part of the kinetic term be given by:

$$L_{KE}=\dot{q}\ln_{\kappa }\;\left(\dot{q}+\sqrt{{\dot{q}}^{2}+1}\right)=\dot{q}\frac{{\left(\dot{q}+\sqrt{{\dot{q}}^{2}+1}\right)}^{\kappa }-1}{\kappa }.$$

We now compute the equation of motion using the Tsallis deformed kinetic energy.

**Proposition** **9.** *The Euler–Lagrange equation of the Tsallis κ-deformed Lagrangian*

(90) $$L_{\kappa }^{RT}=\dot{q}\ln_{\kappa }\;\left(\dot{q}+\sqrt{{\dot{q}}^{2}+1}\right)-\frac{1}{2}\left(\dot{q}+\sqrt{{\dot{q}}^{2}+1}+\frac{1}{\dot{q}+\sqrt{{\dot{q}}^{2}+1}}\right)-\gamma^{-1}\cosh\;q$$

*yields*

(91) $$\frac{\ddot{q}}{\sqrt{{\dot{q}}^{2}+1}}{\left(\dot{q}+\sqrt{{\dot{q}}^{2}+1}\right)}^{\kappa }+\left(\frac{\dot{q}}{\sqrt{{\dot{q}}^{2}+1}}{\left(\dot{q}+\sqrt{{\dot{q}}^{2}+1}\right)}^{\kappa }-1\right)+\gamma^{-1}\sinh\;q=0.$$

## 5. Outlook

In this paper we considered a special class of Lagrangians proposed by Calogero and Leyvraz with an “exotic” kinetic energy term. This term has a close resemblance to the Shannon entropy function, $\dot{q}\ln\;\dot{q}$. Using this new type of Lagrangian, we derived the celebrated Lotka–Volterra and replicator equation. We then generalized the construction of Calogero and Leyvraz and considered a different type of kinetic energy term based on cross entropy. We then manufactured an N=2 relativistic Toda lattice system. We also discussed the significance of this equation in modern physics. Different avatars of this equation appeared in string theory and theoretical high energy physics—purely imaginary position and momentum coordinates lead to the Hofstadter model.

The main goal is to express all these celebrated equations in terms of logarithmic kinetic energy using the deformation of the entropic kinetic energy term. We used the Kaniadakis κ-deformed logarithm and exponential functions to deform these Calogero–Leyvraz type Lagrangians to give a new formulation of κ-deformed Lotka–Volterra, replicator equation and N=2 relativistic Toda lattice system. We also extended this deformation to the Tsallis class and derived Tsallis-deformed equations. All the original equations can be recovered from the deformed systems when κ→0.

In a nutshell, this paper elucidated the strength of the Calogero–Leyvraz formalism based on entropic kinetic terms. It would be interesting to derive more known and not so well known systems using this method. The predator–prey models are one of the best places to apply our scheme. We may try to apply this scheme to planar generalized Lotka–Volterra (GLV) equations; for example, consider two interacting populations with densities x>O and y>O with the simplest formal description of interaction with the linear dependence of the growth rates $\dot{x}/x$ and $\dot{y}/y$. This yields the following GLV equation:

$$\dot{x}=x\left(a+bx+cy\right),\;\dot{y}=y(d+ex+fy).$$

We can generalize this construction and check whether we can manufacture this new equation using the Calogero–Leyvraz formalism. We then implement the κ-deformation of such equations and study their dynamics.

It would be worth investigating the Calabi–Yau manifold connected to the κ-deformed Hamiltonian of the N=2 relativistic Toda lattice equation.

## Data Availability

This article does not use data. No new data have been created or analysed in the present manuscript.
